# First Case of *Chryseobacterium gleum* Post-COVID-19 in a Child with Recurrent Fever

**DOI:** 10.3390/reports7040090

**Published:** 2024-11-02

**Authors:** Manuela Colosimo, Filippo Luciani, Maria Novella Pullano, Diana Marisol Abrego-Guandique, Luca Gallelli

**Affiliations:** 1 Department of Microbiology and Virology, AO Dulbecco: Pugliese Hospital, 88100 Catanzaro, Italy; manuelacolosimo@hotmail.it; 2 Infectious Disease Ambulatory, Department of Prevention, ASP, 87100 Cosenza, Italy; filippoluciani@gmail.com; 3 Department of Pediatry, AO Dulbecco: Pugliese Hospital, 88100 Catanzaro, Italy; novellapullano@gmail.com; 4 Department of Health Science, School of Medicine, 88100 Catanzaro, Italy; dianamarisol.abregoguandique@unicz.it; 5 Department of Health Science, School of Medicine, Research Center FAS@UNICZ, University of Catanzaro, 88100 Catanzaro, Italy; 6 Operative Unit of Clinical Pharmacology, Dulbecco University Hospital, 88100 Catanzaro, Italy

**Keywords:** child, microbiology, *Chryseobacterium gleum*, isolation, MALDI-TOF

## Abstract

**Background and Clinical Significance:** *Chryseobacterium gleum* is a Gram-negative opportunistic and emerging pathogen able to induce systemic manifestations (e.g., peritonitis, pneumonia, urinary tract infections, meningitis) in immunocompromised patients. No data on children have been published. **Case Presentation:** A 2-year-old child presented in the pediatric ambulatory room with recurrent fever, submandibular lymphadenopathy, and skin rash. Laboratory findings revealed the presence of microcytic anemia with an increase in c-reactive protein. Chest X-ray reported mild accentuation of the bronchial structure, especially on the right side and middle–lower zone. In the peripheral blood smear, anisopoikilocytosis and elliptical red cells were evident. Clinical evaluation revealed the presence of conjunctivitis and polymorphic erythema, hyperemic pharynx and tonsils, SPO2 99%, auscultation of the chest, harsh vesicular murmur all over the area, and some wheezing. Microbiological analysis of sputum and throat swabs revealed the presence of numerous colonies of *Chryseobacterium gleum* confirmed using matrix-assisted laser desorption/ionization time-of-flight mass spectrometry (MALDI-TOF MS score > 2.2). **Conclusions:** This is the first case of *Chryseobacterium gleum* post-COVID in a child. We suggest that a quick identification and an appropriate treatment represent the critical factors able to prevent the adverse outcomes related to *C. gleum* infection.

## 1. Introduction and Clinical Significance

*Chryseobacterium gleum* (*C. gleum*) is an aerobic, Gram-negative, rod-shaped, non-motile bacterium, catalase-positive, oxidase-positive, and indole-positive pathogen that is able to hydrolyze starch and reduce nitrates and nitrites. It is found in the environments (e.g., soils, plant roots, flowers, freshwater) [1,2,3] and nosocomially in submandibular support devices (e.g., ventilators, central lines) [2]. *C. gleum* represents a potential opportunistic and emerging pathogen. Even if it does not cause serious infections in humans, it could induce systemic manifestations (e.g., peritonitis, pneumonia, urinary tract infections, meningitis) in immunocompromised patients or indwelling catheters [4,5,6,7,8,9,10,11,12,13]. This report details the case of a young, immunocompetent child who presented with recurrent fever infected by *C. gleum*.

## 2. Case Description

**Figure 1:**
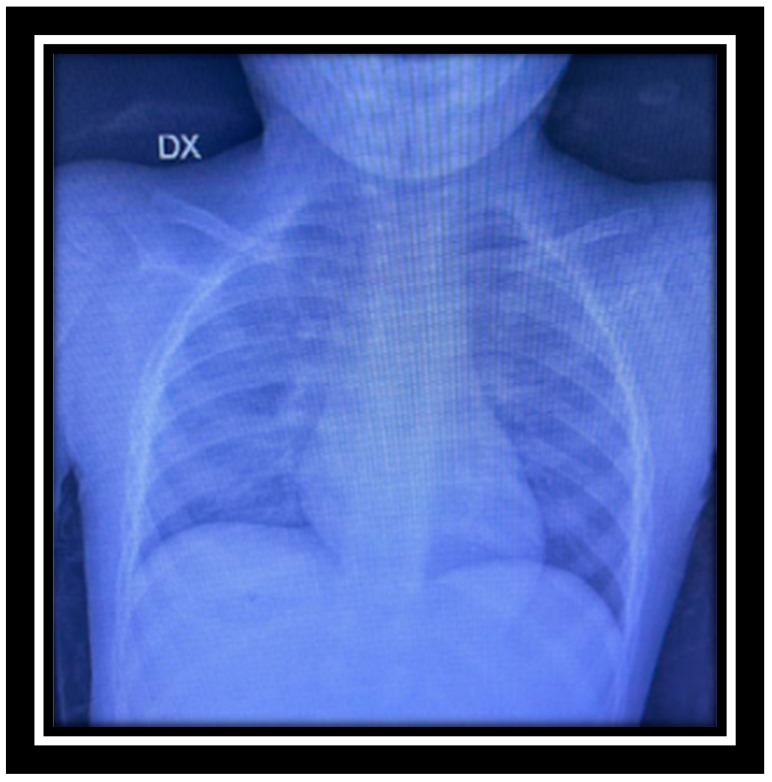
Chest X-ray.

A 2-year-old child with a history of COVID-19 infection six months ago presented to the pediatric outpatient clinic with recurrent fever, submandibular lymphadenopathy, and a skin rash. Laboratory findings revealed the presence of microcytic anemia with an increase in c-reactive protein. At pediatric admission, a throat swab was performed, and blood agar, MacConkey agar (MCK), Sabouraud and Columbia Nalidixic Acid (CNA) plates were used. It is left to incubate for 24–48 h at 37 °C except for CNA, which is preserved in CO_2_. In the first 24 h, numerous yellow colonies were highlighted on the blood agar and MCK plates, which were identified with the MALDI-TOF MS system as *C. gleum*. A further throat swab and sputum examination were requested. The bacterium grew on both samples in the first 24 h and was again identified with the mass spectrometry identification system. An ultrasound of the neck and abdomen, in conjunction with a hematological evaluation, led to the diagnosis of submandibular lymphadenomegaly and anisopoikilocytosis. Additionally, the dermatologist diagnosed atopic dermatitis and prescribed topical treatment comprising a glucocorticoid (hydrocortisone 17-butyrate) and acetaminophen. At the follow-up, one month later, due to the persistence of symptoms, he was hospitalized in the pediatric division. A dermatological evaluation follow-up confirmed the presence of atopic skin disease, while an otorhinolaryngology consultation diagnosed tonsillar hypertrophy without ongoing inflammation. During the hospitalization, laboratory tests, bone marrow fine needle aspiration, and a new neck and abdomen ultrasound failed to reveal a systemic disease. In detail, chest X-ray reported mild accentuation of the bronchial structure, especially on the right side and in the middle–lower zone. Free costophrenic sinuses, cardiac shadow within the limits of a regular aorta, several oval lymph nodes with thick and hypoechoic and thin cortex, and hyperechoic central hilum in place, as shown in Figure 1.

In the lymph node echo on the right, in the submandibular area, oval and hypoechoic lymph nodes with vascular spots were visible. The peripheral blood smear exhibited anisopoikilocytosis and elliptical red cells, as well as several activated lymph nodes and some atypical lymphocytes with nucleoli and nuclear notches. Additionally, there were some naked nuclei and monocytes with different cytoplasmic tingabilities. The fine needle aspiration of the marrow demonstrated high cellularity (C3, M1, MGK present), with infiltrations that were compatible with the subject’s age. The myeloid series was present, as were notes of erythropoiesis in the erythroid series. In the microcytic anemia testing, also known as complete blood count, the carrier of beta thalassemia trait, as well as molecular analysis for triplication of the negative alpha gene, was present. The patient was negative for thrombophilia (see summary in Table 1).

Moreover, an increase in erythrocyte sedimentation rate was documented in Table 2.

**Figure 2:**
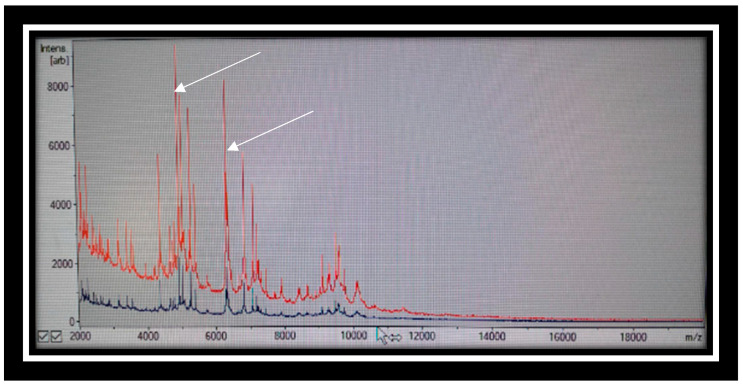
*C. gleum*. identification using matrix-assisted laser desorption ionization time-of-flight mass spectrometry (MALDI-TOF MS). Red line represents the sample of the child; blue line represents a reference sample (negative control). Each peak in the spectra represents proteins of a specific charge and size. The white arrow indicates the *C. gleum*.

Clinical evaluation revealed the presence of conjunctivitis and polymorphic erythema. An infectious disease was postulated, and clarithromycin was prescribed. Two weeks later (14 March 2024), due to the persistence of symptoms, he was transferred to another operative unit of child diseases, where he developed a fever (39.5 °C). Clinical evaluation revealed the presence of conjunctivitis and polymorphic erythema. The clinical evaluation reported hyperemic pharynx and tonsils, SPO2 99%, auscultation of the chest, harsh vesicular murmur all over the area, and some wheezing. Microbiological analysis of sputum and throat swabs revealed the presence of numerous colonies of *C. gleum*. According to the Bartlett scheme, the confirmed swab and the sputum examination were positive after the suitability assessment. Matrix-assisted laser desorption/ionization time-of-flight mass spectrometry (MALDI-TOF MS score > 2.2) was used for the pathogen’s confirmation test, as is shown in Figure 2.

The Sensitive system by Sensititre Thermo scientific™ Gram-negative MIC plate was used to evaluate the antibiotic sensitivity (Table 3). The system follows the EUCAST interpretation.

## 3. Discussion

*Chryseobacterium* was identified for the first time as a medically significant bacterial genus, making up 0.27% of non-fermentative Gram-negative bacilli collected from samples across 16 countries [14]. *C. gleum* was the least common (two isolates—4%). *C. gleum* infection in humans affects immunosuppressed individuals and device carriers, hence it is a pathological opportunist in the hospital. The highest prevalence was found among the elderly [15]. Six cases of *C. gleum* infection were reported during 2012–2015 and one in 2018 [16,17]. For instance, a case report highlighted *C. gleum* as the agent of pneumonia in an adult male with diffuse large B-cell lymphoma, illustrating its potential to cause severe respiratory infections in vulnerable populations [15]. Additionally, *C. gleum* has been implicated in urinary tract infections and bloodstream infections, further emphasizing its clinical significance [12,18]. This pathogen does not frequently infect the pediatric population; however, a case published in 2016 from Saudi Arabia reported *C. gleum* pneumonia in a 6-month-old baby with nephrotic syndrome [9].

Post-COVID-19, there has been an increase in reports in the literature of infections with *C. gleum* [19]. Prolonged hospitalizations due to COVID-19, especially those requiring mechanical ventilation, are associated with an increased risk of healthcare-associated infections caused by rare and opportunistic organisms, such as *C. gleum*. Angrup et al. described 18 *C. gleum* isolates that were identified from 10 patients, with 17 of these isolates found in 9 COVID-19-positive patients, suggesting a possible association between COVID-19 and *C. gleum* respiratory tract infections [20].

Therefore, to the best of our knowledge and according to literature review, this is the first case of *C. gleum* reported in a post-COVID-19 pediatric patient. This pathogen is especially concerning due to its intrinsic resistance spectrum to carbapenems, aminoglycosides, and combinations of beta-inhibitors lactams/beta-lactamases and cephalosporins, commonly used to treat sepsis [15]. However, some studies have reported susceptibility to fluoroquinolones and tetracyclines, indicating that treatment options may be limited but not entirely absent [19,21]. The resistance mechanisms, including the production of beta-lactamases, further complicate the management of infections caused by *C. gleum* [15]. In this case report, antibiotic resistance was found in the following antibiotics: amikacin, amoxyclav aztreonam, cefotaxime, ertapenem, gentamicin, meropenem, piperacillin/tazobactam, and tobramycin (Table 3). Due to extremely limited sensitivity, there is a risk that *C. gleum* could become a severe infectious threat. Despite its scarcity, it is imperative that medical professionals recognize potential risk factors and maintain clinical suspicion of *C. gleum*, when appropriate. Respect for hand hygiene, aseptic procedures, and timely identification and treatment are all critical factors in preventing adverse outcomes related to *C. gleum* infection. The patient fully recovered after pharmacological therapy with ceftazidime antibiotics.

## Figures and Tables

**Table 1:** Clinical laboratory and radiological evaluation during the admission.

Examination	Results
Chest X-ray	mild accentuation of the bronchial structure
Lymph node echo	oval lymph nodes with thick, hypoechoic and thin cortex and hyperechoic central hilum
Peripheral blood smear	anisopoikilocytosis and elliptical red cells
Fine needle aspiration of the marrow	rich cellularity (C3, M1, MGK present) infiltrate
Microcytic Anemia Testing ^§^	carrier of beta thalassemia trait, molecular analysis for triplication of the negative alpha gene

C3: atypical cells in shape; M1: macrophages; MGK: megakaryocytes; ^§^ molecular biology.

**Table 2:** Blood laboratory tests.

	Admission (8 March)	Follow-Up (19 March)	Normal Range
White Blood Cells	9.99	19.68	4.60–10.20 × 10^3^/mm^3^
Red Cells	5.47	5.47	4.20–6.10 × 10^6^/µL
Hemoglobin	8.5	8.8	12–18 g/dL
Hematocrit Test	29.6	29.6	37–52% of red blood cells
Mean Corpuscular Volume	54.1	54.1	80–99 µm^3^
Mean Corpuscular Hemoglobin	16	16	27–32 pg/cell
Mean Corpuscular Hemoglobin Concentration	29.6	29.6	32–37 g/dL
Platelets	424	325	130–400
Mean Platelet Volume	7	7	7.2–11
Neutrophils	12.12 (61.6%)	12.12 (61.6%)	10.63–6.96 (39.3–73.7%)
Lymphocytes	6.11 (31.1%)	6.11 (31.1%)	1.09–2.99 (18–48%)
Monocytes	1.22 (6.19%)	1.22 (6.19%)	0.24–0.79 (4.4–12.7% of white blood cells)
Eosinophils	-	-	0.03–0.44 (0.6–7.3% of white blood cells)
Basophils	0.23 (1.2%)	0.23 (1.2%)	0.0–0.08 (0.0–1.7% of white blood cells)
Erythrocyte Sedimentation Rate	107	107	<20 mm/h
C-reactive Protein	4.88	188	0.3–5 mg/L
Procalcitonin	0.02	1.11	once 0.05 ng/ml
EBV			
VCA IgM		19	U/mL > 40
VCA IgG		>750	U/mL > 40
*Bartonella henselae*			
IgM		0.156	Index > 1.1
IgG		0.785	Index > 1.1

**Table 3:** Antibiogram evaluation of sputum culture.

Antibiotics	MIC	Sensitivity Pattern
Amikacin	32	Resistant
Amoxyclav	64	Resistant
Aztreonam	32	Resistant
Cefotaxime	8	Resistant
Ceftazidime	2	Susceptible
Ceftazidime/avibactam	<0.5	Susceptible
Ceftalozane/tazobactam	<0.5	Susceptible
Ciprofloxacin	0.25	Susceptible
Ertapenem	2	Resistant
Gentamicin	8	Resistant
Imipenem	4	Intermediate
Meropenem	16	Resistant
Piperacillin/tazobactam	32	Resistant
Tigecycline	0.5	Susceptible
Tobramycin	8	Resistant

## Data Availability

The original data presented in this study are available on reasonable request from the corresponding author. The data are not publicly available due to privacy.
